# Safety and effectiveness of HSK21542 for hemodialysis patients: a multiple ascending dose study

**DOI:** 10.3389/fphar.2023.1203642

**Published:** 2023-10-09

**Authors:** Mingming Pan, Guihua Wang, Li Zhou, Yan Xu, Li Yao, Chaoqing Wu, Changlin Mei, Zhanzheng Zhao, Dong Sun, Tianjun Guan, Qinkai Chen, Ming Shi, Hui Xu, Weifang Zeng, Fangqiong Li, Rui Yan, Bi-Cheng Liu

**Affiliations:** ^1^ Department of Nephrology, Zhong Da Hospital, Southeast University School of Medicine, Nanjing, China; ^2^ Division of Nephrology, Kidney Research Institute, West China Hospital, Sichuan University, Chengdu, China; ^3^ Department of Nephrology, The Affiliated Hospital of Qingdao University, Qingdao, China; ^4^ Department of Nephrology, The First Hospital of China Medical University, Shenyang, China; ^5^ Department of Nephrology, The People’s Hospital of Guangxi Zhuang Autonomous Region, Nanning, China; ^6^ Division of Nephrology, The Second Affiliated Hospital Navy Medical University, Shanghai, China; ^7^ Department of Nephrology, The First Affiliated Hospital of Zhengzhou University, Zhengzhou, China; ^8^ Department of Nephrology, Affiliated Hospital of Xuzhou Medical University, Xuzhou, China; ^9^ Division of Nephrology, Zhongshan Hospital, Xiamen University, Xiamen, China; ^10^ Department of Nephrology, The First Affiliated Hospital of Nanchang University, Nanchang, China; ^11^ Department of Nephrology, Renmin Hospital of Wuhan University, Wuhan, China; ^12^ Division of Nephrology, Xiangya Hospital of the Central South University, Changsha, China; ^13^ Sichuan Haisco Pharmaceutical Co., Ltd., Chengdu, China; ^14^ Department of Nephrology, Affiliated Hospital of Guizhou Medical University, Guiyang, China

**Keywords:** CKD-aP, HSK21542, hemodialysis, κ-opioid receptor agonist, uremic pruritus

## Abstract

**Background:** HSK21542, a novel selective peripherally-restricted κ-opioid receptor agonist has been proven to be a safe and effective analgesic and antipruritic drug in both *in vitro* and *in vivo* studies. We aimed to evaluate its safety, pharmacokinetics and efficacy in hemodialysis patients over a 1-week treatment period, and to establish the optimal dosage for a further 12-week stage 2 trial.

**Methods:** In this multiple ascending dose study, hemodialysis patients were randomly assigned to receive HSK21542 (0.05–0.80 μg/kg), or a placebo three times within 2.5 h at the end of each dialysis session for 1 week. Safety evaluations included reports of treatment-emergent adverse events (TEAEs); pharmacokinetics and efficacy outcomes were also assessed.

**Results:** Among the 44 screened patients, 41 were enrolled and completed the trial. The overall incidence of TEAEs was higher in the HSK21542 group compared to the placebo group, with an incidence of 75.0%, 50.0%, 75.0%, and 88.9% in the range of 0.05–0.80 μg/kg. All TEAEs were grade 1 or 2 in severity. HSK21542 exhibited linear pharmacokinetics characteristics within the dose range 0.05–0.80 μg/kg, without drug accumulation after multiple-doses. Compared to the placebo, a significant decrease of the weekly mean Worst Itching Intensity Numerical Rating Scale was found in the HSK21542-0.30 μg/kg group (*p* = 0.046), but without significant improvement in the Skindex-16 score.

**Conclusion:** HSK21542 was well tolerated in the dose range 0.05–0.80 μg/kg in hemodialysis patients. HSK21542-0.3 μg/kg exhibited promising efficacy in patients with moderate to severe pruritus and warrants a further Stage 2 trial.

**Clinical Trial Registration:** https://clinicaltrials.gov/, identifier NCT04470154.

## 1 Introduction

End-stage renal disease (ESRD) is characterized by accompanying systemic life-threatening complications even when treated with replacement therapies (Yang et al., 2020). Up to 50% of hemodialysis patients experience itching and about 40% of them have pruritus, with degrees of suffering ranging from moderate to severe (Pisoni et al., 2006). The condition has been termed chronic kidney disease-associated pruritus (CKD-aP). Moderate-to-severe pruritus not only directly affects the quality-of-life (QoL) of patients, such as their poor quality of sleep or even depression in severe cases, but also causes skin damage and infections induced by repeated scratching (Pisoni et al., 2006; Mathur et al., 2010; Kimata et al., 2014). Moreover, CKD-aP is associated with high mortality in hemodialysis patients due to an increased risk of inflammation, infection and depression (Pisoni et al., 2006; Yamamoto et al., 2009; Kimata et al., 2014).

Currently, the treatment options for CKD-aP vary based on different pathogenesis, such as dialysis (Ko et al., 2013), ultraviolet-B (UVB) light therapy (Ko et al., 2011) or xerosis treatment, typically prescribed for mild pruritus (Young et al., 2009), whereas systematic drug therapy including antihistamines is commonly used as first-line therapy but often has as poor clinical efficacy (Amirkhanlou et al., 2016). More recently, a new alternative therapy has been developed by targeting the endogenous opioid system through blockade of μ-opioid receptors (MOR) and/or activation of κ-opioid receptors (KOR) (Cowan et al., 2015), drugs such as KOR-nalfurafine hydrochloride (Kumagai et al., 2010) and difelikefalin (Fishbane et al., 2020a) or the MOR-naltrexone (Pauli-Magnus et al., 2000), which revealed their potential in relieving the itching of hemodialysis patients.

HSK21542, is a novel selective peripherally-restricted KOR agonist, that cooperatively regulates potassium and calcium currents involved in G protein activation, thus achieving the anti-nociceptive and antipruritic effects by blocking pain and itch signal transduction, inhibiting the excitability of dorsal root ganglia and peripheral sensory nerves, thus reducing the release of inflammatory factors and neurotransmitters (Violin et al., 2014; Wang et al., 2021). In the first-in-human phase 1 clinical trials performed in Australians (ACTRN12619001739101, 0.02∼1.5 μg/kg) and Chinese (NCT04110886, 0.2∼3.375 μg/kg) healthy subjects, HSK21542 was shown to be well tolerated and exhibited linear pharmacokinetic (PK) characteristics, with no dose-dependent trend in the elevation of serum prolactin concentrations.

Based on the aforementioned findings, a multicenter, randomized, double-blind, placebo-controlled phase 2 trial was conducted to determine the safety, pharmacokinetic properties and clinical efficacy of HSK21542 in patients undergoing hemodialysis. The phase 2 trial adopted a 2-Stage design and the present study only reported the results of the Stage 1 trial, which was a multiple ascending dose study conducted in hemodialysis patients who received over 1-week multiple intravenous administrations of HSK21542. Referring to the dosage of HSK21542 used in the phase 1 trial and the similar KOR agonist difelikefalin, the dose range of HSK21542 was set at 0.05–0.80 μg/kg in the present Stage 1 trial to define the optimal doses for use in a subsequent Stage 2 trial, which was conducted over a 12-week administration period.

## 2 Materials and methods

### 2.1 Design of the study and the enrolled patients’ characteristics

This was a multicenter, double-blind, randomized, placebo-controlled phase 2 trial that adopted a 2-Stage design, which only reports the results of the Stage 1 period. The Stage 1 trial was a dose-escalation trial conducted in hemodialysis patients who received over 1-week multiple intravenous administrations of HSK21542. The main objective was to establish the safety and tolerability of HSK21542 after 1-week of its intravenous administration to hemodialysis patients. Secondary objectives were to determine the PK characteristics of HSK21542 and the drug exposure correlation of serum prolactin concentrations, as well as the efficacy outcomes in patients undergoing hemodialysis suffering from moderate-to-severe pruritus.

ESRD patients aged between 18 and 75 years (inclusive), regardless of sex, who received hemodialysis (including hemodiafiltration) 3 times a week for ≥3 months before screening were included. New York Heart Association (NYHA) patients rated ≥ Class III during the screening period or patients with confirmed electrocardiograph (ECG) abnormalities were ineligible for enrollment (including QTcF ≥480 ms) as judged by the investigator; patients who were expected to have a kidney transplant and/or parathyroidectomy; patients who had a history of opioid allergies, such as hives, or had used opioids in the week prior to screening or could not avoid using opioids other than the study drugs during the entire study period were also excluded. Inclusion and exclusion criteria are presented in more detail in Supplementary Material S1.

The institutional review board of the Clinical Research of Zhongda Hospital Affiliated to Southeast University approved the study protocols (Approval No. 2020ZDSYLL112-P06) as did the Affiliated Hospital of Guizhou Medical University (Approval No. 2020077) and all other participating centers. Written inform consent was provided by all patients enrolled in the study, which was registered at ClinicalTrials.gov (NCT04470154).

### 2.2 Study procedure and assessment

Stage I included the screening, administration observations and the follow-up times. Forty patients receiving hemodialysis were scheduled to be randomly assigned to HSK21542 (0.05, 0.15, 0.30, 0.80 μg/kg) and placebo groups in a ratio of 4:1, with 8 patients per group. Each patient received one dose only, and the HSK21542 or placebo was administered intravenously within 2.5 h after the end of each hemodialysis session on Days 1, 3 and 5, respectively, i.e., 3 times in a 1-week administration period. HSK21542 or placebo were administered through the arm without a fistula, or via a non-fistula vessel of the arm with fistula, in which drug administration and PK blood sampling could not be performed on the ipsilateral arm.

According to the dose-escalation principle, patients were started on a low dose of 0.05 μg/kg, and after all 8 patients had received the last dose and completed a 7-day safety assessment, then the next dose (0.15 μg/kg) was given to the next 8 patients, and so on for subsequent dose groups until the maximum dose of 0.80 μg/kg was administered. During the study, dose adjustment or early discontinuation of dose-escalation could be made according to the safety, tolerability, PK and/or pharmacodynamics (PD) results. Criteria for early discontinuation of the dose-escalation included, but were not limited to: ≥ grade 2 drug-related adverse events (AEs) in ≥1/2 patients or ≥ grade 3 drug-related AEs in ≥1/3 patients in the dose group.

Pruritus intensity was evaluated via a 24-h Worst Itching Intensity Numerical Rating Scale (WI-NRS), with scores ranging from 0 to 10, where 10 indicated the worst itching intensity (Phan et al., 2012). No less than 4 patients with moderate to severe degrees of pruritus were enrolled in each dose group, which was defined as those with at least one 24-h WI-NRS score ≥4 at screening admission, Day-1 or prior to drug administration on Day 1 (baseline). QoL was evaluated through a Skindex-16 score, ranging from 0 to 100, that assessed 3 categories related to itch (symptoms, emotions and functioning); higher scores indicated a worse QoL (Zhang, 2013). Patients with moderate to severe pruritus were required to complete the WI-NRS and Skindex-16 scale during the screening, before and 24 h (±2 h) after each dose at the end of each dialysis session but before the fourth dialysis (±4 h). Blood samples (3 mL) for PK analysis were collected 30 min before the first dose (after the end of dialysis), immediately, 1, 6, and 24 h after the first dose, prior to the second and third dialysis, 30 min before the third dose (after the end of dialysis), immediately, 1, 6, and 24 h after the third dose, and prior to the fourth dialysis. The blood samples used for the detection of serum prolactin concentrations were collected before the first dose (after the end of dialysis), 1 h after the first dose, prior to the third dialysis, 1 h after the third dose and prior to the fourth dialysis. All AEs were coded based on the Medical Dictionary for Regulatory Activities (MedDRA) and AE severity was graded according to the Common Terminology Criteria for Adverse Events (CTCAE, ver. 5.0).

### 2.3 Study endpoints

#### 2.3.1 Safety endpoints

Reports of AEs and serious AEs, laboratory tests (routine blood/urine, blood biochemistry, thyroid and coagulation functions), vital signs (heart and respiration rates, diastolic and systolic blood pressure measurements, temperature and blood oxygen saturation [SpO_2_]) levels and a 12-lead ECG examination were documented. In addition, the correlation between the serum prolactin concentration and HSK21542 drug exposure in hemodialysis patients was evaluated.

#### 2.3.2 PK endpoints

1) The plasma concentration-time curve after single and multiple doses of HSK21542 in those patients undergoing hemodialysis; 2) PK parameters after single and multiple doses of HSK21542, including the area under the plasma drug concentration-time curve (AUC_0-t_, AUC_0-∞_), the maximum plasma concentration (C_max_), time to C_max_ (t_max_), half-life time (t_1/2_), clearance (CL) and the apparent volume of distribution (V_ss_). In addition, the trough concentration (C_min_), accumulation ratio based on C_max_ and AUC only were calculated after multiple doses had been administered.

#### 2.3.3 Efficacy endpoints

Weekly changes from baseline for 24-h daily WI-NRS and the Skindex-16 score in patients with pruritus.

### 2.4 Sample sizes and randomization

The estimation of sample sizes was not based on statistical considerations. Forty patients were scheduled to be enrolled, and the sample size of each group was set to 10 patients, including 8 patients who received HSK21542 and 2 who received a placebo. The study was designed in a double-blind set, with non-blinded researchers responsible for the administration of the study drug, strictly ensuring that the investigator, patients and the entire operation of the study were all blinded. The randomized block method was performed through the Interactive Web Response System (IWRS), which assigned eligible patients to HSK21542 and placebo groups in a ratio of 4:1; the corresponding random and drug numbers were generated using SAS software (ver. 9.4).

### 2.5 Statistical analysis

Statistical analyses were conducted using SAS (ver. 9.4). Variables that were continuous are given as the mean ± SD or the median with ranges (minimum, maximum), while categorical variables are presented as numbers and percentages. The full analysis set (FAS) considered all the randomized patients who received HSK21542 or a placebo, and had one or more assessable efficacy indicators, that were employed for demographic, baseline characteristics and efficacy outcome analyses. The PK analysis set (PKS) included all-randomized patients who had been given HSK21542, and had at least one assessable plasma concentration and PK parameter. Safety and serum prolactin concentration analyses after medication were conducted in all randomized patients who received HSK21542 or placebo, who had at least one available data value about safety indicators and the prolactin concentration, respectively. The Wilcoxon rank-sum test was used to compare the weekly changes from baseline for efficacy outcomes between HSK21542 and the placebo groups, such as 24-h daily WI-NRS and the Skindex-16 score. All other indicators are presented using descriptive statistics in the Stage 1 trial. A two-sided *p*-value <0.05 was considered to be statistically significant.

## 3 Results

### 3.1 Patients and baseline characteristics

**FIGURE 1:**
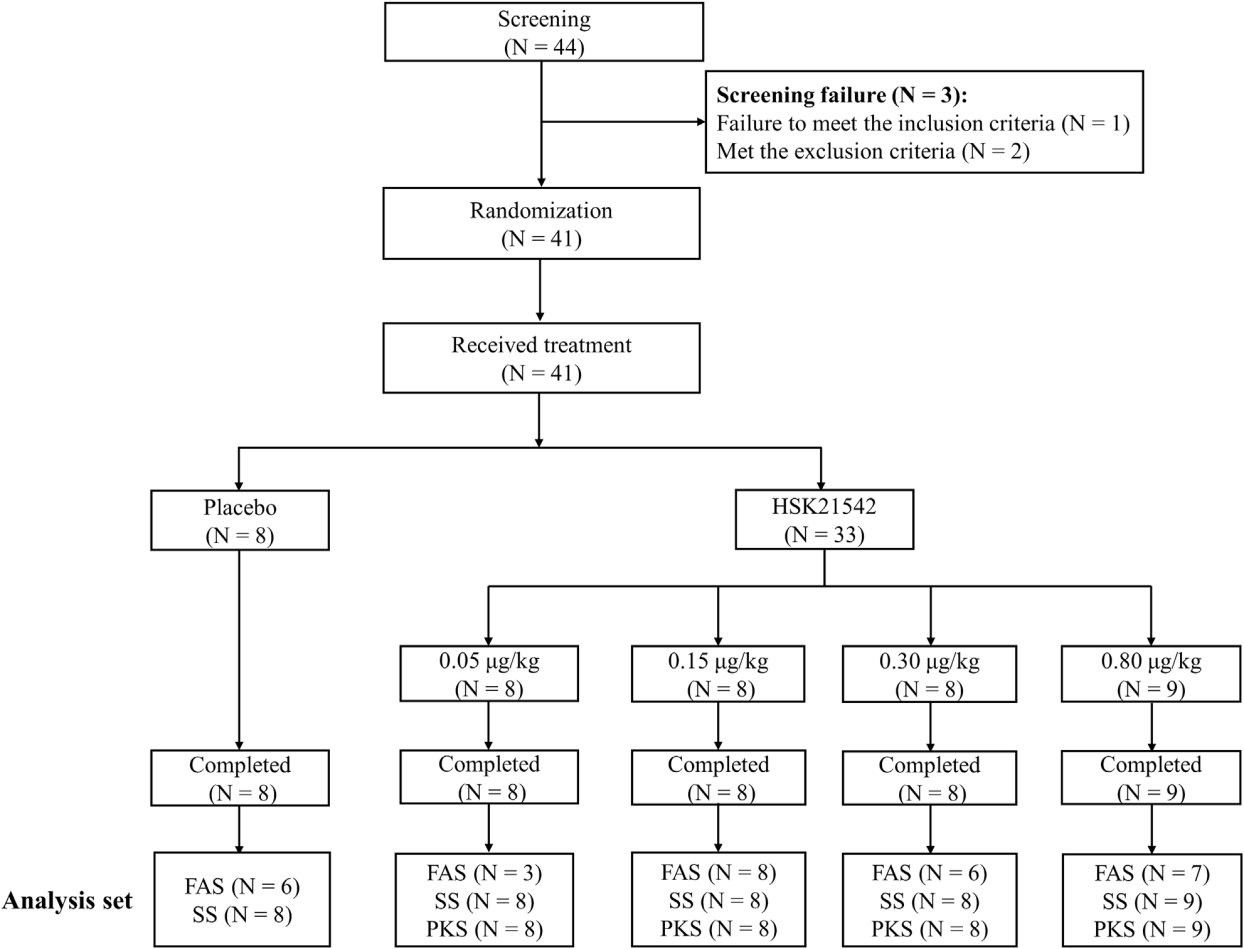
Patients’ disposition in the Stage 1 trial Abbreviation: FAS, full analysis set; PKS, pharmacokinetics analysis set; SS, safety set.

Among the 44 screened patients, except for 1 who did not meet the inclusion criteria and 2 who met the exclusion criteria, a total of 41 patients were enrolled and completed the trial across 11 centers in China from 15 September 2020 to 10 August 2021 (Figure 1). A total of 41 patients (including 8 patients in the placebo group and 33 patients in the HSK21542 group) were analyzed for safety and their serum prolactin concentrations, and 33 in the HSK21542 group were included in the PKS. Thirty patients with moderate to severe pruritus were included in the FAS, with 6, 3, 8, 6, and 7 in the placebo and HSK21542-0.05, 0.15, 0.30, and 0.80 μg/kg groups, respectively.

The demographic and baseline characteristics are shown in Supplementary Table S1. The patients’ age range was between 29 and 74 years, and there were more males than females in each group. The baseline itch intensity in the majority of patients was moderate to severe in the placebo group (100%) and in the HSK21542 treatment group (91.7%).

### 3.2 Safety

A total of 67 AE occurred in 26 patients across all groups which were TEAEs. The overall incidence of TEAEs in the HSK21542 groups was higher than in the placebo group (72.7% vs. 25.0%), with an incidence of 75.0%, 50.0%, 75.0%, and 88.9% in the range of 0.05–0.80 μg/kg (Table 1). All TEAEs were grade 1 or grade 2 in severity. The overall incidence of drug-related TEAEs in the HSK21542 groups was comparable to the placebo group (30.3% vs. 25.0%), with an incidence of 37.5%, 12.5%, 37.5%, and 33.3% in the 0.05, 0.15, 0.30, and 0.80 μg/kg dose groups, respectively. No serious AEs (SAEs) or deaths were reported, or any TEAEs which led to drug discontinuation or patient withdrawal from the study.

**TABLE 1:** Summary of AEs during the Stage 1 period (SS).

	Placebo (n = 8)	HSK21542				
		0.05 μg/kg (*n* = 8)	0.15 μg/kg (*n* = 8)	0.30 μg/kg (*n* = 8)	0.80 μg/kg (*n* = 9)	Total (*n* = 33)
Any AEs, n (%)	2 (25.0)	6 (75.0)	4 (50.0)	6 (75.0)	8 (88.9)	24 (72.7)
Any TEAEs, n (%)	2 (25.0)	6 (75.0)	4 (50.0)	6 (75.0)	8 (88.9)	24 (72.7)
Grade 1	0	4 (50.0)	2 (25.0)	4 (50.0%)	4 (44.4)	14 (42.4)
Grade 2	2 (25.0)	2 (25.0)	2 (25.0)	2 (25.0%)	4 (44.4)	10 (30.3)
Drug-related TEAEs, n (%)	2 (25.0)	3 (37.5)	1 (12.5)	3 (37.5)	3 (33.3)	10 (30.3)
Grade 2	1 (12.5)	0	0	0	1 (11.1)	1 (3.0)
Any SAEs, n (%)	0	0	0	0	0	0
Any TEAEs leading to the drug discontinuation or withdraw from the trial, n (%)	0	0	0	0	0	0
Death	0	0	0	0	0	0
Most frequently reported TEAEs (≥2 patients) in any treatment group, termed by PT, n (%)						
Intradialytic hypotension	0	3 (37·5)	1 (12·5)	2 (25·0)	2 (22·2)	8 (24·2)
Dizziness	0	2 (25·0)	0	1 (12·5)	3 (33·3)	6 (18·2)
Hypotension	0	2 (25·0)	0	1 (12·5)	0	3 (9·1)
Lower abdominal pain	0	0	0	2 (25·0)	0	2 (6·1)
Drug-related TEAE, termed by PT, n (%)						
Dizziness	0	1 (12·5)	0	0	2 (22·2)	3 (9·1)
Hypotension	0	2 (25.0)	0	1 (12·5)	0	3 (9·1)
Paresthesia	1 (12·5)	0	0	1 (12·5)	1 (11·1)	2 (6·1)
Lower abdominal pain	0	0	0	2 (25·0)	0	2 (6·1)
Headache	0	0	1 (12·5)	0	0	1 (3·0)
Sensation disorders	0	0	0	0	1 (11·1)	1 (3·0)
Abdominal pain	0	0	0	0	1 (11·1)	1 (3·0)
Palpitations	0	0	0	0	1 (11·1)	1 (3·0)
Gastritis	1 (12·5)	0	0	0	0	0

Note. AEs, adverse events; PT, preferred terms; SAEs, serious adverse events; SS, safety set; TEAEs, treatment-emergent adverse events.

Among the most common TEAEs, intradialytic hypotension (37.5%) and hypotension (25.0%) were more frequently reported in the HSK21542-0.05 μg/kg group, while dizziness was more prevalent in the HSK21542-0.80 μg/kg group (33.3%), followed by the HSK21542-0.05 μg/kg group (25.0%). The most common reported drug-related TEAEs in the HSK21542 groups were hypotension, paresthesia and dizziness, which occurred more often in the 0.05, 0.30, and 0.80 μg/kg groups, in 2 patients in each group. Lower abdominal pain was only found in 2 (25.0%) patients in the HSK21542-0.3 μg/kg group. Most drug-related TEAEs were grade 1. Grade 2 drug-related TEAEs only occurred in 1 patient in the placebo group (12.5%, gastritis) and HSK21542-0.80 μg/kg group (11.1%, abdominal pain), separately. The majority of TEAEs were self-limiting or were alleviated after brief clinical treatment.

As shown in Supplementary Figure S1, compared with the placebo, no significant dose correlation was found for changes in the serum prolactin concentrations after patients had received HSK21542 for 1-week. There was no significant correlation between the HSK21542 plasma and serum prolactin concentrations. Compared with baseline, there were no notable clinical differences in laboratory test results, vital signs, 12-ECG or physical examinations after HSK21542 administration, with comparable profiles found in the placebo group.

### 3.3 Plasma concentration and PK parameters

**FIGURE 2:**
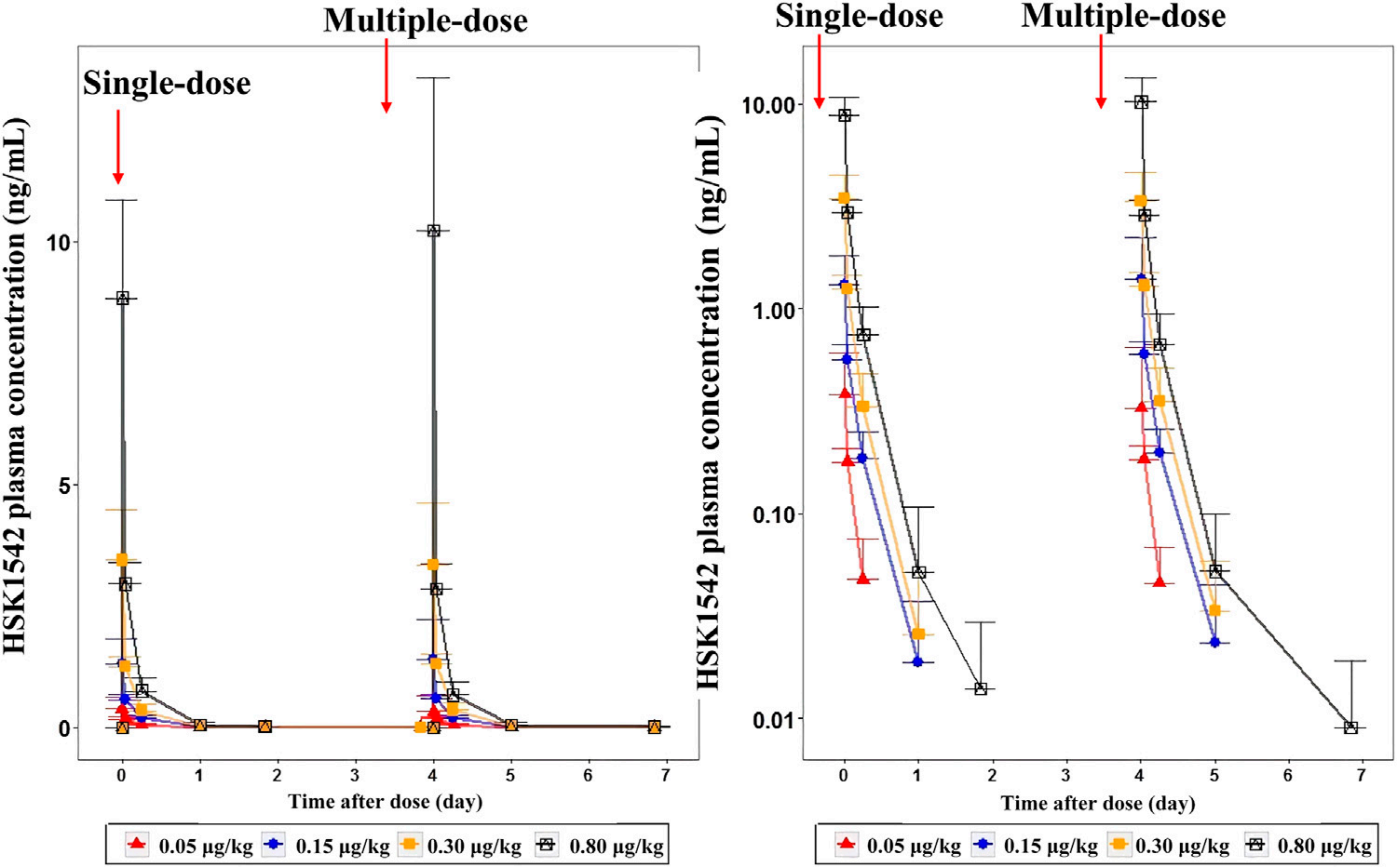
Mean plasma concentration-time curves after single- and multiple-intravenous injection of HSK21542 in each dose group during the Stage 1 period (left: linear, right: semi-log).

A dose-dependent increase in the plasma concentration was observed after single and multiple doses of HSK21542 were administered. The maximum plasma concentration was reached at about 0.03–0.05 h after the intravenous injection of HSK21542 (Figure 2). The drug exposure (C_max_ and AUC) of HSK21542 increased with the dose increase within the range 0.05∼0.80 μg/kg (Table 2). The mean RacC_max_ and RacAUC of HSK21542 were 0.94–1.09 and 0.96–1.19, respectively.

**TABLE 2:** Pharmacokinetics parameters after single and multiple doses of HSK21542 in each dose group during the Stage 1 period.

PK parameters	HSK21542			
	0.05 μg/kg	0.15 μg/kg	0.30 μg/kg	0.80 μg/kg
Single dose				
C_max_ (ng/mL)	0.40 ± 0.21 (51%)	1.31 ± 0.49 (38%)	3.44 ± 1.03 (30%)	9.27 ± 1.72 (19%)
T_max_ (h)	0.03 (0.03, 1.03)	0.05 (0.04, 1.00)	0.05 (0.03, 0.07)	0.05 (0.04, 0.06)
AUC_0−t_ (ng·h/mL)	0.91 ± 0.45 (50%)	3.82 ± 1.57 (41%)	7.63 ± 2.88 (38%)	17.40 ± 3.29 (19%)
AUC_0−∞_ (ng·h/mL)	1.20 ± 0.58 (48%)	4.25 ± 1.49 (35%)	8.57 ± 2.92 (34%)	17.50 ± 3.18 (18%)
t_1/2_ (h)	3.57 ± 2.35 (66%)	5.21 ± 2.07 (40%)	4.97 ± 2.19 (44%)	4.29 ± 1.47 (34%)
CL (L/h)	2.64 ± 0.93 (35%)	2.71 ± 1.37 (51%)	2.49 ± 0.93 (37%)	3.07 ± 1.04 (34%)
V_ss_ (L)	11.70 ± 2.57 (22%)	15.60 ± 3.02 (19%)	12.90 ± 2.23 (17%)	12.60 ± 3.02 (24%)
Multiple dose				
C_max_ (ng/mL)	0.39 ± 0.25 (64%)	1.43 ± 0.79 (55%)	3.34 ± 1.27 (38%)	10.10 ± 3.32 (33%)
T_max_ (h)	0.04 (0.03, 1.03)	0.05 (0.04, 1.03)	0.04 (0.03, 0.06)	0.05 (0.04, 0.06)
AUC_0−t_ (ng·h/mL)	0.87 ± 0.40 (46%)	4.27 ± 1.55 (36%)	8.49 ± 3.09 (36%)	17.00 ± 4.95 (29%)
AUC_0−tau_ (ng·h/mL)	1.28 (0.86, 1.69)	4.71 ± 1.12 (24%)	8.53 ± 2.87 (34%)	17.70 ± 4.63 (26%)
AUC_0−∞_ (ng·h/mL)	1.28 (0.86, 1.71)	4.79 ± 1.29 (27%)	7.21 ± 2.57 (36%)	14.50 ± 1.83 (13%)
t_1/2_ (h)	4.10 (2.08, 6.13)	5.82 ± 2.82 (48%)	3.65 ± 1.39 (38%)	3.71 ± 0.45 (12%)
CL (L/h)	2.40 (1.72, 3.09)	2.22 ± 0.71 (32%)	2.76 ± 0.92 (33%)	3.42 ± 0.76 (22%)
V_ss_ (L)	12.60 (9.34, 15.9)	15.20 ± 3.99 (26%)	10.90 ± 1.42 (13%)	13.00 ± 1.02 (8%)
RacC_max_	0.94 ± 0.22 (24%)	1.09 ± 0.43 (39%)	1.01 ± 0.25 (25%)	1.07± 0.24 (22%)
RacAUC	0.96 (0.92, 1.01)	1.19 ± 0.32 (27%)	1.00 ± 0.05 (5%)	1.00 ± 0.18 (18%)

Note. Data are presented as the mean ± SD (CV%) or the median with ranges (minimum, maximum).

Abbreviation: AUC, area under curve; CV, coefficient of variation; RacCmax, accumulation ratio based on C_max_; RacAUC, accumulation ratio based on AUC; PK, pharmacokinetics; SD, standard deviation.

### 3.4 Efficacy

**FIGURE 3:**
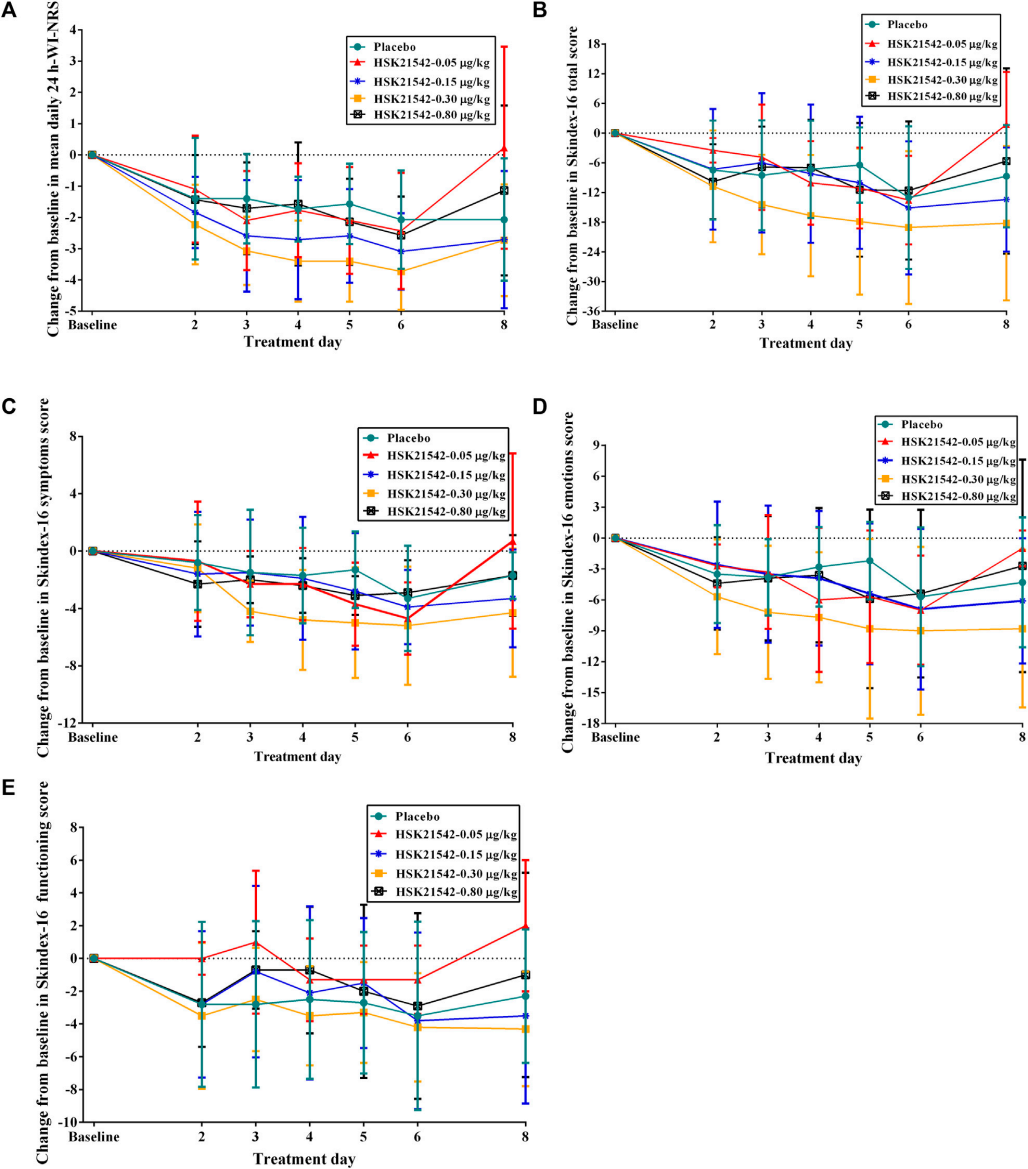
Trends of changes from baseline in the **(A)** mean of daily 24-h WI-NRS and Skindex-16 based on **(B)** total score, **(C)** symptoms score, **(D)** emotions score and **(E)** functioning score during 1-week administration of HSK21542 or placebo. Abbreviation: WI-NRS, Worst Itching Intensity Numerical Rating Scale.

The daily 24-h WI-NRS decreased in patients with moderate to severe pruritus across all groups during 1-week treatment, in which, patients in the HSK21542-0.15 and 0.30 μg/kg groups exhibited greater reduced changes from baseline than the placebo group (Figure 3A). Of note, a significant reduction from the first doses in the weekly mean WI-NRS was only found in the HSK21542-0.30 μg/kg group, in comparison to the placebo group (−3.10 ± 0.93 vs. −1.72 ± 1.31, *p* = 0.046) (Table 3). Compared to the placebo group, a clear improvement from baseline towards Skindex-16 total, symptoms, emotions and functioning scores were shown throughout the 1-week period in the HSK21542-0.30 μg/kg group (Figures 3B–D). However, the apparent weekly decreased changes in Skindex-16 scores from first dosing were not significantly different in the HSK21542-0.30 μg/kg and placebo groups (all *p* > 0.05).

**TABLE 3:** Weekly changes from baseline for various efficacy outcomes in all groups.

Efficacy endpoints	Placebo (*n* = 6)	HSK21542				
		0.05 μg/kg (*n* = 3)	0.15 μg/kg (*n* = 8)	0.30 μg/kg (*n* = 6)	0.80 μg/kg (*n* = 7)	Total (*n* = 24)
Weekly mean of daily 24−h WI−NRS						
Baseline	5.57 ± 0.86	5.77 ± 1.86	6.71 ± 1.25	5.90 ± 1.27	4.86 ± 0.95	5.85 ± 1.39
Weekly changes from first dosing	−1.72 ± 1.31	−1.57 ± 1.82	−2.59 ± 1.33	−3.10 ± 0.93	−1.79 ± 1.37	−2.35 ± 1.36
*p*-value vs. placebo		0.845	0.078	0.046	0.804	0.131
Skindex-16 total score						
Baseline	43.42 ± 13.74	41.33 ± 10.63	49.63 ± 19.63	29.70 ± 22.17	29.17 ± 21.06	37.64 ± 20.97
Weekly changes from first dosing	−8.58 ± 9.60	−6.88 ± 6.92	−10.02 ± 12.38	−16.18 ± 12.77	−8.75 ± 10.52	−10.80 ± 11.23
*p*-value vs. placebo		0.714	0.950	0.310	0.836	0.860
Skindex-16 symptoms score						
Baseline	11.20 ± 4.17	11.70 ± 4.04	12.10 ± 2.36	7.20 ± 2.93	6.90 ± 1.77	9.30 ± 3.51
Weekly changes from first dosing	−1.72 ± 2.76	−2.17 ± 3.06	−2.46 ± 3.40	−4.12 ± 3.26	−2.40 ± 1.71	−2.82 ± 2.83
*p*-value vs. placebo		0.905	0.755	0.240	0.650	0.502
Skindex-16 emotions score						
Baseline	20.80 ± 9.75	20.70 ± 6.81	21.60 ± 8.86	13.30 ± 12.86	13.90 ± 11.87	17.20 ± 10.79
Weekly changes from first dosing	−3.72 ± 4.47	−4.27 ± 4.54	−4.73 ± 6.46	−7.87 ± 6.97	−4.31 ± 6.88	−5.33 ± 6.30
*p*-value vs. placebo		0.964	0.573	0.260	0.445	0.743
Skindex-16 functioning score						
Baseline	9.70 ± 7.55	7.30 ± 3.06	13.90 ± 9.08	8.00 ± 7.56	7.30 ± 7.32	9.70 ± 7.85
Weekly changes from first dosing	−2.78 ± 4.58	−0.17 ± 1.89	−2.39 ± 4.60	−3.57 ± 2.87	−1.66 ± 3.94	−2.19 ± 3.70
*p*-value vs. placebo		0.548	0.950	0.288	>1.000	0.811

Note. Data are presented as the median with ranges (minimum, maximum). *p*-values were derived using the Wilcoxon rank-sum test. Abbreviation: WI-NRS, worst itching intensity numerical rating scale.

## 4 Discussion

A dose-escalation Stage 1 trial was conducted in hemodialysis patients who received HSK21542 intravenously three times for a 1-week period at the end of each dialysis session. The preliminary results revealed that HSK21542 was well tolerated by patients in doses ranging from 0.05 to 0.80 μg/kg. No SAEs, deaths or any TEAEs which lead to drug discontinuation or patient withdrawal from the study were reported.

Selective KOR agonists do not have the common side effects elicited by MOR agonists, such as tolerance, addiction, respiratory depression and constipation, but often trigger central nervous system (CNS)-mediated side effects such as hallucinations, sedation, diuresis and dysphoria (Viscusi et al., 2021; Shram et al., 2022). It is well known that peripherally-restricted KOR agonists have low penetration across the blood-brain barrier, thus CNS-mediated side effects are avoided to a certain extent but the analgesic and antipruritic effects are retained (Albert-Vartanian et al., 2016; Paton et al., 2020). In the present study, the most commonly reported TEAEs (≥2 patients) in patients treated with HSK21542 were intradialytic hypotension, hypotension, dizziness and lower abdominal pain, among which, intradialytic hypotension was not related to the HSK21542 administration. In addition to hypotension and dizziness, the most frequently previously reported TEAEs, such as insomnia, somnolence, diarrhea, nasopharyngitis, nausea and vomiting, were absent or rarely reported in patients who received HSK21542 (Mathur et al., 2010; Fishbane et al., 2020b; Fishbane et al., 2022; Narita et al., 2022). In addition, only 2 patients in the HSK21542-0.30 μg/kg group developed lower abdominal pain, which was possible associated with HSK21542 administration, but with a severity of grade 1, and the symptoms disappeared without any treatment. Of note, no significant dose correlation was found for changes in the serum prolactin concentration, and no associated hyperprolactinemia was reported after patients received 1-week HSK21542, contrary to the actions of nalfurafine hydrochloride (Kumada et al., 2017). Overall, HSK21542 exhibited excellent safety outcomes in the present study, which should be confirmed in future long-term studies.

In agreement with data obtained in a previous study in healthy volunteers, HSK21542 exhibited linear PK characteristics after a single dose or 1-week of multiple doses in hemodialysis patients, and that C_max_ and AUC increased proportionally with dose escalation. In addition, no drug accumulation occurred after multiple-doses of HSK21542, with a lower accumulation ratio, findings comparable to those reported for other KOR agonists (Deeks, 2021; Miyamoto et al., 2022). However, HSK21542 was rapidly eliminated from the plasma and exhibited a shorter t_1/2_ compared to that reported for other KOR agonists (Deeks, 2021; Miyamoto et al., 2022).

In terms of efficacy, the antipruritic effect and QoL improvement after 1-week HSK21542 medication were preliminarily explored in moderate to severe hemodialysis patients. Compared to placebo, only patients in the HSK21542-0.30 μg/kg group exhibited significantly decreased changes from baseline in the weekly mean WI-NRS, whereas the Skindex-16 score did not shown a significant reduction in all domains examined. It is inevitable that the placebo effect is a known confounder in double-blind clinical trials related to itching assessment, because the itching-related scale is insensitive, with strong individual subjectivity (van Laarhoven et al., 2015). Moreover, considering the short treatment period and the small sample size, the preliminary efficacy results can only confidently be used as a reference for a subsequent Stage 2 trial involving a larger cohort of patients. Another limitation of the present study was that serum calcium and phosphate concentrations were not measured, but they will be measured in a subsequent Stage 2 trial along with the incidence of related TEAEs, such as hypocalcemia and hypercalcemia.

In conclusion, hemodialysis patients treated with HSK21542 exhibited good tolerance in the dose range 0.05–0.80 μg/kg, without significant changes in the serum prolactin concentration. HSK21542 had linear PK characteristics after a single dose or 1-week multiple-dose administration in hemodialysis patients, without drug accumulation occurring. HSK21542-0.30 μg/kg dosage is likely to be promising therapy for hemodialysis patients with moderate to severe pruritus and warrants a further stage 2 trial.

## Data Availability

The raw data supporting the conclusion of this article will be made available by the authors, without undue reservation.
