# Supplementary material for: Safety and effectiveness of HSK21542 for hemodialysis patients: a multiple ascending dose study
Source: Front Pharmacol. 2023 Oct 9;14:1203642. doi: 10.3389/fphar.2023.1203642 (PMC10590914; doi:10.3389/fphar.2023.1203642)
Supplement: Supplementary file 1 [file DataSheet1.pdf]

Supplementary Materials

Safety and effectiveness of HSK21542 for hemodialysis patients: a multiple ascending dose study

Mingming Pan[#], Guihua Wang[#], Li Zhou, Yan Xu, Li Yao, Chaoqing Wu, Changlin Mei, Zhanzheng Zhao, Dong Sun, Tianjun Guan, Qinkai Chen, Ming Shi, Hui Xu, Weifang Zeng, Fangqiong Li, Rui Yan[*] and Bi-Cheng Liu[*]

[#]These authors contributed equally to this work and share first authorship.

[*]Correspondence: Bi-Cheng Liu: liubc64@163.com; Rui Yan: yryr1234@126.com

Supplementary Table 1. Demographic and baseline characteristic of patients in Stage 1 trial (FAS)

	**Placebo (n = 6)**	**HSK21542**				
		**0.05 μg/kg (n = 3)**	**0.15 μg/kg (n = 8)**	**0.30 μg/kg (n = 6)**	**0.80 μg/kg (n = 7)**	**Total (n = 24)**
Age (years)	47.7 ± 11.4	34.0 ± 5.6	54.9 ± 10.6	43.5 ± 7.2	45.4 ± 10.6	46.7 ± 11.2
Gender, n (%)						
Male	5 (83.3)	2 (66.7)	6 (75.0)	5 (83.3)	5 (71.4)	18 (75.0)
Female	1 (16.7)	1 (33.3)	2 (25.0)	1 (16.7)	2 (28.6)	6 (25.0)
Height (cm)	164.8 ± 5.4	164.3 ± 9.1	168.0 ± 5.5	164.7 ± 6.0	164.1 ± 8.1	165.6 ± 6.7
Dry weight (kg)	62.7 ± 7.6	58.2 ± 5.0	65.2 ± 9.5	67.0 ± 9.4	62.4 ± 12.0	63.9 ± 9.7
BMI (kg/m^2)	23.1 ± 2.7	21.5 ± 0.8	23.1 ± 2.9	24.7 ± 2.9	23.1 ± 3.9	23.3 ± 3.1
Smoking, n (%)						
Never	4 (66.7)	2 (66.7)	5 (62.5)	2 (33.3)	2 (28.6)	11 (45.8)
Previous	2 (33.3)	0	1 (12.5)	0	3 (42.9)	4 (16.7)
Current	0	1 (33.3)	2 (25.0)	4 (66.7)	2 (28.6)	9 (37.5)
Drinking, n (%)						
Never	6 (100)	3 (100)	4 (50.0)	6 (100)	4 (57.1)	17 (70.8)
Previous	0	0	3 (37.5)	0	0	6 (25.0)
Current	0	0	1 (12.5)	0	3 (42.9)	1 (4.2)
Worst itching intensity, n (%)						
Mild	0	1 (33.3)	0	0	1 (14.3)	2 (8.3)
Moderate	5 (83.3)	1 (33.3)	4 (50.0)	5 (83.3)	6 (85.7)	16 (66.7)
Severe	1 (16.7)	1 (33.3)	4 (50.0)	1 (16.7)	0	6 (25.0)

Note. Data are presented as the median with ranges (minimum, maximum) or numbers with percentages.
BMI, body mass index; WI-NRS, Worst Itching Intensity Numerical Rating Scale

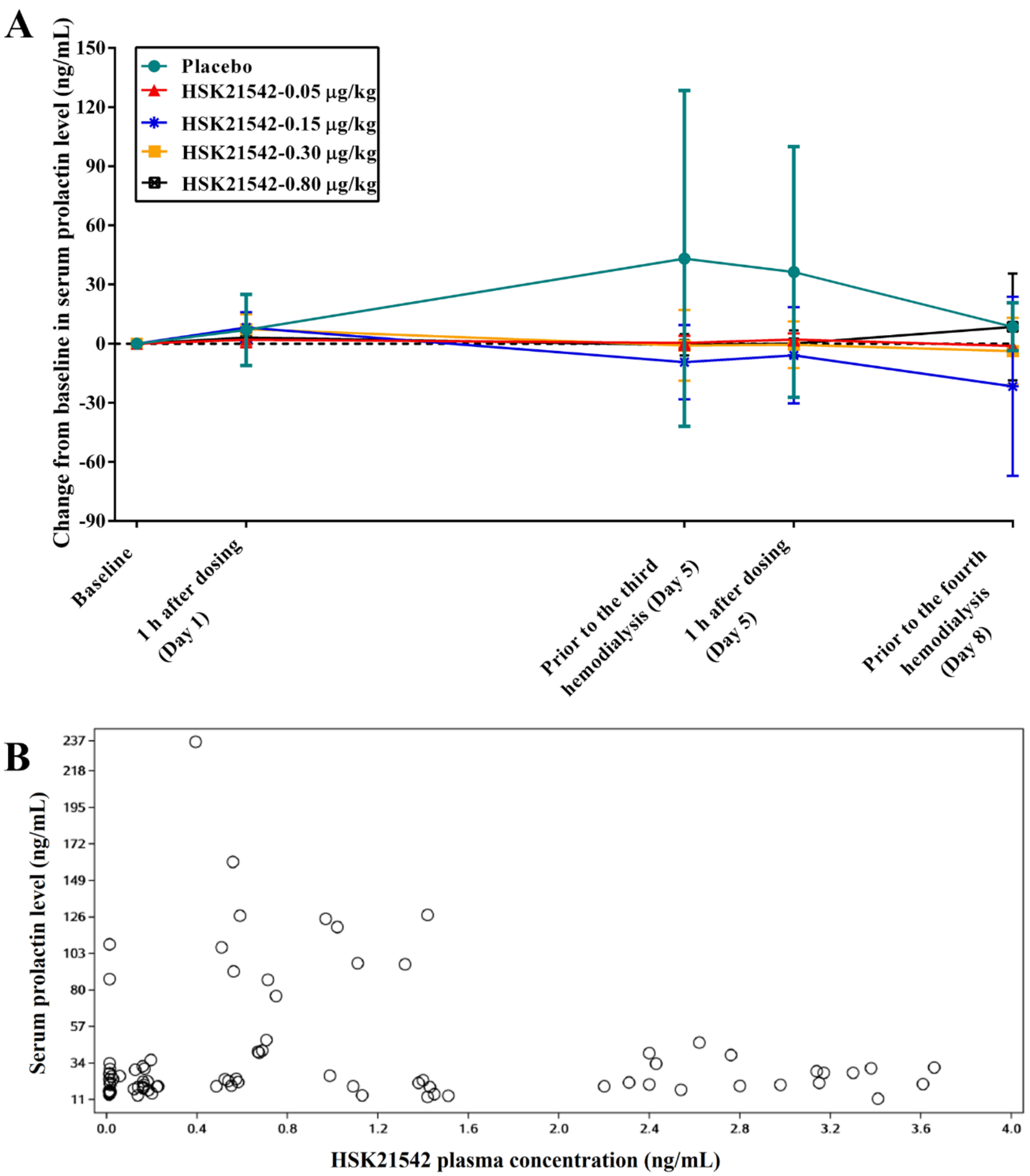

Supplementary Figure 1. (A) Changes in the serum prolactin concentration from baseline in the placebo and HSK21542 groups; (B) Correlation between plasma concentration of HSK21542 and serum prolactin concentrations during 1-weeks administration.

Supplementary File 1. Inclusion and exclusion criteria for the Stage 1 trial

1. Inclusion criteria

All of the following criteria had to be met:

1) The patient was willing to sign an informed consent form, fully understood the objectives and purpose of the study, and were willing to comply with the study protocol before any of the study-related procedures started;
2) Aged ≥ 18 and ≤ 75 years old, male or female;
3) Dry weight ≥ 50.0 kg at screening, body mass index (BMI) in the range of 16.0–30.0 kg/m^2 (inclusive);
4) Patients with end-stage renal disease who received hemodialysis (including hemodiafiltration) 3 times per week prior to screening for at least 3 months;
5) Patients with at least 2 occurrences of single-pool urea clearance index (sp Kt/V) ≥ 1.2, or at least 2 occurrences of urea reduction ratio (URR) ≥ 65%, or 1 occurrence of sp Kt/V ≥ 1.2 and 1 occurrence of URR ≥ 65% on different days of dialysis within 3 months before screening;
6) Male subjects must have agreed to use condoms during sexual intercourse for the duration of the study and for 3 months after the last study dose; female subjects should have had the menopause for at least 1 year, or should have had permanent sterilization (e.g., fallopian tube occlusion, hysterectomy, bilateral salpingectomy); women of childbearing potential had to agree to take effective contraceptive measures during the study and within 3 months after the last drug administration, such as oral contraceptives, condoms or a contraceptive diaphragm.

2. Exclusion criteria

Patients were excluded if any one of the following criteria were met:

1) Expected to undergo kidney transplantation and/or parathyroidectomy during the study;
2) History of allergy to opioids, such as urticaria (note: adverse effects related to opioid use, such as constipation and nausea were not included as exclusion criteria in this study);
3) Used opioids within 1 week before screening, or were unable to avoid the use of opioids other than the investigational drug during the study;
4) Participated in any clinical trial of other drug or medical device study within 1 month of screening (e.g., received study medication or were treated by a medical device in a clinical trial);
5) Had a blood perfusion within 3 months before screening;
6) History of medication or drug abuse;
7) Average daily alcohol consumption > 15 g (15 g of alcohol is equivalent to 450 mL of beer or 150 mL of wine or 50 mL of light liquor) within 3 months before screening;
8) Unable to comply with the standard dietary plan and unable to avoid coffee or tea during the study period;
9) Blood pressure of upper limbs in the supine position at screening: systolic blood pressure < 90 mmHg, diastolic blood pressure < 60 mmHg or systolic blood pressure > 180 mmHg, diastolic blood pressure > 110 mmHg;

10) New York Heart Association (NYHA) Class ≥ III at screening or confirmed to have an abnormal ECG at screening or determined by the investigator to be inappropriate to be enrolled, including QTcF ≥ 480 ms;
11) Alanine aminotransferase (ALT) or aspartate aminotransferase (AST) or total bilirubin ≥ 1.5 × upper limit of normal (ULN) at screening;
12) Blood sodium > 155 mmol/L at screening;
13) Hemoglobin ≤ 80 g/L at screening;
14) Positive for hepatitis B surface antigen (HBsAg), hepatitis C antibody (HCVAb), syphilis antibody, or human immunodeficiency virus (HIV) antibody at screening;
15) Had underwent major surgery (defined by the investigator as major surgery) within 3 months before screening;
16) Had a total blood loss of ≥ 200 mL within 1 month before screening, except for blood loss during menstruation in females;
17) Females who were pregnant or breastfeeding;
18) Any physiological or psychological diseases or conditions that may increase the risk to the patient during the study, affect their compliance with the protocol, or affect their completion of the trial, as judged by the study physician, including but not limited to:
    (a) Severe mental illness or cognitive impairment (e.g., dementia);
    (b) Any other relevant acute or chronic neurological and psychiatric diseases (e.g., encephalopathy, coma, delirium) within 3 months before screening;
    (c) Patients with malignant tumors (including malignant tumors that had been cured without recurrence).
